# Crossing the Interspecies Barrier: Opening the Door to Zoonotic Pathogens

**DOI:** 10.1371/journal.ppat.1004129

**Published:** 2014-06-19

**Authors:** Christian Gortazar, Leslie A. Reperant, Thijs Kuiken, José de la Fuente, Mariana Boadella, Beatriz Martínez-Lopez, Francisco Ruiz-Fons, Agustin Estrada-Peña, Christian Drosten, Graham Medley, Richard Ostfeld, Townsend Peterson, Kurt C. VerCauteren, Christian Menge, Marc Artois, Constance Schultsz, Richard Delahay, Jordi Serra-Cobo, Robert Poulin, Frederic Keck, Alonso A. Aguirre, Heikki Henttonen, Andrew P. Dobson, Susan Kutz, Juan Lubroth, Atle Mysterud

**Affiliations:** 1 SaBio IREC (CSIC-Universidad de Castilla-La Mancha), Ciudad Real, Spain; 2 Department of Viroscience, Erasmus University Medical Centre, Rotterdam, Netherlands; 3 Department of Veterinary Pathobiology, Center for Veterinary Health Sciences, Oklahoma State University, Stillwater, Oklahoma, United States of America; 4 Center for Animal Disease Modeling and Surveillance, Department of Medicine and Epidemiology, University of California, Davis, Davis, California, United States of America; 5 Faculty of Veterinary Medicine, Department of Animal Pathology, University of Zaragoza, Zaragoza, Spain; 6 Universitäts-Klinikum Bonn und Medizinische Fakultät, Bonn, Germany; 7 School of life Sciences, University of Warwick, Coventry, United Kingdom; 8 Harvard Center for the Environment, Harvard University, Cambridge, Massachusetts, United States of America; 9 Biodiversity Institute University of Kansas, Lawrence, Kansas, United States of America; 10 USDA/APHIS/WS/National Wildlife Research Center, Fort Collins, Colorado, United States of America; 11 Friedrich-Loeffler-Institut, Federal Research Institute for Animal Health, Jena, Germany; 12 Université de Lyon, Campus Vétérinaire de Lyon, VetAgro Sup, Marcy L'Étoile, France; 13 Academic Medical Centre, University of Amsterdam, Amsterdam Institute for Global Health and Development, Amsterdam, Netherlands; 14 National Wildlife Management Centre, Animal Health and Veterinary Laboratories Agency, Nympsfield, Stonehouse, Gloucestershire, United Kingdom; 15 IRBIO and Departement de Biologia Animal, Facultat de Biologia, Universitat de Barcelona, Barcelona, Spain; 16 Department of Zoology, University of Otago, Dunedin, New Zealand; 17 Centre National de la Recherche Scientifique, Laboratoire D'anthropologie Sociale, Paris, France; 18 Department of Environmental Science and Policy, George Mason University, Fairfax, Virginia, United States of America; 19 Vantaa Unit, Finnish Forest Research Institute, Vantaa, Finland; 20 Department of Ecology and Evolutionary Biology, Princeton University, Princeton, New Jersey, United States of America; 21 Department of Ecosystem and Public Health, Faculty of Veterinary Medicine, University of Calgary, Calgary, Canada; 22 Animal Production and Health Division, Food and Agriculture Organization of the United Nations–Headquarters, Rome, Italy; 23 Centre for Ecological and Evolutionary Synthesis (CEES), Department of Biosciences, University of Oslo, Oslo, Norway; Columbia University, United States of America

## Zoonotic Pathogens

The number of pathogens known to infect humans is ever increasing. Whether such increase reflects improved surveillance and detection or actual emergence of novel pathogens is unclear. Nonetheless, infectious diseases are the second leading cause of human mortality and disability-adjusted life years lost worldwide [1], [2]. On average, three to four new pathogen species are detected in the human population every year [3]. Most of these emerging pathogens originate from nonhuman animal species.

Zoonotic pathogens represent approximately 60% of all known pathogens able to infect humans [4]. Their occurrence in humans relies on the human-animal interface, defined as the continuum of contacts between humans and animals, their environments, or their products. The human-animal interface has existed since the first footsteps of the human species and its hominin ancestors 6–7 million years ago, promoting the prehistoric emergence of now well-established human pathogens [5]. These presumably include pathogens with roles in the origin of chronic diseases, such as human T-lymphotropic viruses and *Helicobacter pylori*, as well as pathogens causing major crowd diseases, such as the smallpox and measles viruses and *Bordetella pertussis* [5], [6]. Since prehistory, the human-animal interface has continued to evolve and expand, ever allowing new pathogens to access the human host and cross species barriers [5].

## Species Barriers

The suitability of any species to act as a host to a particular pathogen varies due to both host species– and pathogen-dependent factors, which define the species barriers. The species barriers separating nonhuman animal species from humans and thus of concern for zoonotic pathogens are the focus of this paper. However, the proposed conceptual framework is applicable to any host-pathogen system.

The species barriers separating nonhuman animal species from humans represent a major hurdle for effective exposure to, infection by, and subsequent spread of zoonotic pathogens among humans [7]. Accordingly, these species barriers can be divided into three largely complementary sets. First, the interspecies barrier determines the nature and level of human exposure to zoonotic pathogens. Second, the intrahuman barrier determines the ability of zoonotic pathogens to productively infect a human host and effectively cope with the immune response. Third, the interhuman barrier determines the ability of zoonotic pathogens to efficiently transmit among humans, causing outbreaks, epidemics, or pandemics. Zoonotic pathogens may cross, more or less efficiently, one or more of these sets of barriers. Only pathogens that cross all barriers have the potential to sustainably establish in the human population.

Identifying the factors allowing pathogens to cross each of these three sets of barriers is essential to mitigate burdens of known and future emerging zoonotic pathogens. The interspecies barrier, by its nature, involves ecological processes driving animal and human population dynamics and interspecies contact. Prior attempts to define these factors or drivers started as early as 1992 [8]. Recent contributions in this field underlined the importance of landscape change and ecological alteration (e.g., [9]–[11]). Here, we build on these earlier studies to focus on identifying the factors affecting the interspecies barrier from a more holistic perspective, with the aim of developing a simple framework that classifies factors into a limited number of mutually exclusive categories acting at distinct spatial and temporal scales.

## Conceptual Framework for Pathogen Emergence at the Interspecies Barrier

**Figure 1:**
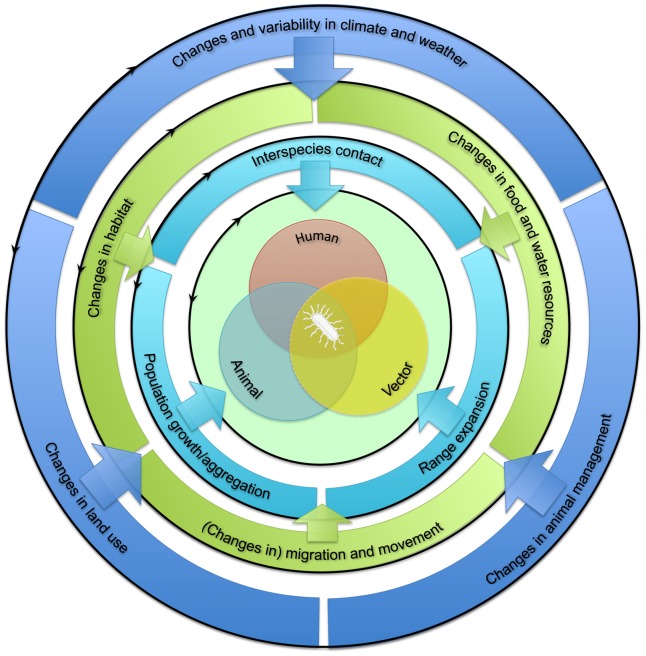
Framework for the classification of drivers of human exposure to animal pathogens (interspecies barrier). See text for more details.

The emergence of zoonotic pathogens in humans is dependent on interactions between humans and infected animal reservoir and/or vector hosts or their environment (Figure 1, center). The extent of such interactions is influenced by the prevalence of zoonotic pathogens in the animal reservoir or vector populations, which is in turn influenced by these populations' health and immune status. In addition, the population dynamics of humans, animal reservoirs, and vectors drive ecological processes that govern pathogen abundance and spread, both within and among species [12]. Increased exposure of humans to animal pathogens can result from changes in the dynamics of any of these populations (Figure 1, inner circle). These changes can be divided into three categories: first, increased interspecies contact between humans and the animal reservoir and/or vector; second, population growth or aggregation of humans, animal reservoir, and/or vector; and third, their geographic range expansion, at least where this expansion involves overlapping ranges. Changes in one aspect of human, animal reservoir, or vector population dynamics may affect another; for example, population growth may accompany range expansion.

Factors influencing these changes in human, animal reservoir, and vector population dynamics may themselves be divided into two sets of drivers, acting at distinct scales. First, “proximate drivers” occurring at the local landscape scale are direct determinants of changes in human, animal reservoir, and vector population dynamics (Figure 1, middle circle; Table 1). These drivers may include habitat suitability, food and water resource availability, and short- or long-distance movements. The extent to which changes in these proximate drivers affect human, animal reservoir, and vector population dynamics depends greatly on the ecology of the species under consideration. For example, changes in habitat may favor generalist species but may drive specialist species to local extinction.

**Table 1:** Drivers for overcoming the interspecies barrier.

Ultimate Drivers	Proximate Drivers
**Climate variability and change**	**Movement/migration**
El Niño-Southern Oscillation and North Atlantic Oscillation	Displacement (e.g., due to flooding or habitat destruction)
Warming; season extension; extremes in heat and cold	Inhibited migration (e.g., due to fencing)
Flooding	Human urban migration
Drought	**Habitat change**
**Land-use change**	Improved habitat (e.g., for invertebrate vectors due to extension of breeding season)
Deforestation	Loss of natural habitat (e.g., for bats due to deforestation)
Pasture to cropland	Habitat fragmentation
Intensification of crop production	**Food and water change**
Reforestation and agricultural abandonment	Increased food (e.g., for waterbirds due to intensification of crop production or for deer due to winter feeding)
Urbanisation	Changed food (e.g., for dairy cattle due to intensification of livestock production or for humans due to intensification of livestock and crop productions as well as changes in food manufacturing and consumption practices)
**Animal-management change**	Water contamination
*Free-living*	
Changes in harvesting/culling	
Conservation measures and translocations	
Feeding	
Fencing of natural habitat	
*Domestic*	
Intensification of livestock production	
Increasing trade of animals and animal products	

**Figure 2:**
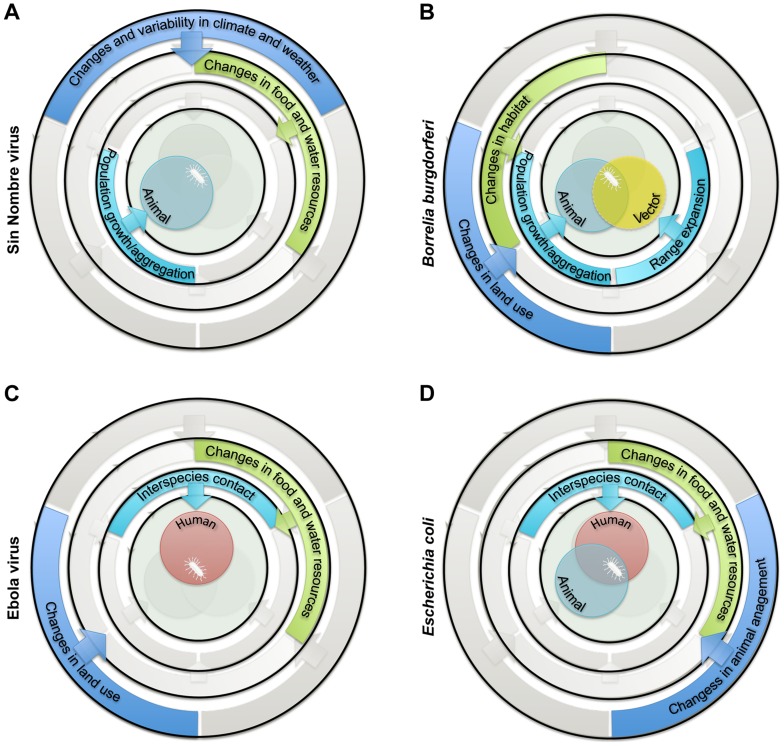
Examples of sets of drivers and ecological processes implicated in the emergence of zoonotic pathogens in humans. (A) Emergence of Sin Nombre virus in the Four Corners area of the United States in 1993 was attributed to population growth of the rodent reservoir (*Peromyscus maniculatus*) following increases in food resources (mast) associated with El Niño-Southern Oscillation events [16]. (B) Emergence of *Borrelia burgdorferi* in the eastern US in 1974 was attributed to population growth and range expansion of white-tailed-deer (*Odocoileus virginianus*) and black-legged-tick (*Ixodes scapularis*) vector populations following increase in suitable habitat due to reforestation and management encouraging high deer densities [17]. (C) Emergence of Ebola virus in the Democratic Republic of Congo (formerly Zaire) in 1976 was attributed to increases in interspecies contact between humans and primates following increases in bushmeat hunting and encroachment into undisturbed habitats [18]. (D) Emergence of enteric pathogenic *Escherichia coli* at the human-animal interface was attributed to increased direct and indirect human-animal contact following changes in the food chain and in water quality, often due to the intensification of livestock production [19].

Second, “ultimate drivers” occurring at broader (regional or global) geographic scales temporally precede and govern changes in proximate drivers (Figure 1, outer circle; Table 1). These drivers include climate, land use, and animal management. Changes in these ultimate drivers may be either anthropogenic (human caused) or “natural.” They can promote changes in one or more proximate drivers. For example, changes in land use may affect both habitat suitability and availability of food and water. Together, the above framework allows the proposed underlying factors affecting the interspecies barrier to be categorized systematically (Figure 2).

## Gaps in Current Knowledge of the Ecology of Zoonotic Pathogens

The proposed framework helps identify essential gaps in our understanding of the chain of emergence of zoonotic pathogens in humans and, in particular, of ecological processes underlying crossing of the interspecies barrier. Major gaps include characterization of the relationships between environmental conditions, especially climate and weather, and host and/or vector population dynamics, as well as exploration of pathogen survival and propagation in the environment. Recent studies have aimed at addressing such issues using novel approaches [13] and are essential in order to detect and predict associations between drivers such as climate change or weather variability and pathogen emergence [14], [15]. The current focus in ecology addresses primarily single host-pathogen systems and needs to be expanded to a multihost, multipathogen perspective. Interactions between host, vector, and pathogenic and nonpathogenic infectious agents likely play important roles in the dynamics of zoonotic pathogens at the human-animal interface [14]. Lastly, systematic assessment of actual human exposure to zoonotic pathogens, e.g., by serology, is lacking, calling for a more holistic approach to understanding the complete chain of emergence. Most evidence for the role of anthropogenic changes, e.g., encroachment into natural habitats, on zoonotic pathogen emergence is anecdotal or indirect and generally biased towards developed countries.

## Future Perspectives

The identification of a limited number of mutually exclusive drivers of zoonotic pathogen emergence and of current knowledge gaps is essential to improve risk assessment and prevention measures. The links between pathogen emergence in humans and their underlying factors are typically speculative and associative and usually only account for a short section of the chain of emergence. Overall, knowledge of causal relationships between changes in population dynamics or interspecies contact, on the one hand, and pathogen emergence in humans, on the other, is fragmentary and incomplete at best. Existing studies in this area generally are limited in scope and typically lack quantitative assessment of human exposure to zoonotic pathogens at the human-animal interface.

The above proposed framework helps in understanding the common mechanisms behind disease emergence by linking pathogen emergence in humans to distinct and well-defined proximate and ultimate drivers. Hence, it may be used to further identify and quantify associations, causal relationships, and risks between ecological changes and pathogen emergence. In the full sense of the One Health concept, it can serve to help optimize efforts to manage disease emergence and spread in the interests of humans, food safety, and biodiversity.
