# Correction: Crossing the Interspecies Barrier: Opening the Door to Zoonotic Pathogens

**DOI:** 10.1371/journal.ppat.1004296

**Published:** 2014-07-08

**Authors:** The PLOS Pathogens Staff

The twenty-first author’s name is misspelled. The correct name is: A. Alonso Aguirre.
